# Becoming comfortable with the uncomfortable: The tale of adaptability

**DOI:** 10.4102/sajp.v79i1.1889

**Published:** 2023-06-13

**Authors:** Anke van der Merwe, Roline Barnes, Mariette Nel

**Affiliations:** 1 School of Health and Rehabilitation Sciences, Faculty of Health Sciences, University of the Free State, Bloemfontein, South Africa; 2 Department of Physiotherapy, Faculty of Health Sciences, University of the Free State, Bloemfontein, South Africa; 3 Department Biostatistics, Faculty of Health Sciences University of the Free State, Bloemfontein, South Africa

**Keywords:** adaptive performance, undergraduate physiotherapy, I-ADAPT measurement, healthcare education, graduate attributes

## Abstract

**Background:**

With the ever-changing healthcare environment and impact of the coronavirus disease 2019 (COVID-19) pandemic on tertiary education, healthcare students need to constantly adapt their approach to learning, clinical practice and well-being. Adaptive performance is therefore vital.

**Objectives:**

To investigate the adaptive performance of final year physiotherapy students at the University of the Free State.

**Method:**

A quantitative descriptive study was performed. All consenting final year undergraduate physiotherapy students registered at the University of the Free State in 2021 were approached for inclusion. The short 55-item I-ADAPT measurement was distributed electronically to all possible participants.

**Results:**

The response rate was 28.5% (*n* = 8). Descriptive statistics, namely frequencies and percentages for categorical data and medians and percentages for numerical data were calculated. The dimensions related to handling work stress (50%), uncertainty (62.2%) and creativity (64.0%) scored the lowest. Emotional response to stress (62.5%) and frustration in response to unpredictable situations (62.5%) was reported.

**Conclusion:**

Uncertainty and unpredictability are inevitable for healthcare students. Stress management and emotional intelligence development are advised for inclusion in undergraduate physiotherapy programmes.

**Clinical implications:**

A need for curricular evaluation to ensure students are equipped with stress management and emotional intelligence skills is proposed.

## Background

With a sudden shift to online course work, limited access to clinical platforms, vastly changing patient clinical presentations and diminishing resources, healthcare students need to constantly adapt their approach to their learning and clinical practice (Fawaz, Hamdan-Mansour & Tassi 2018). Previously, the provision of a variety of clinical placements was viewed as sufficient in order for healthcare students to demonstrate their practical skills and thus theoretical knowledge application (Thurling 2017). However, the changed burden of disease and the coronavirus disease 2019 (COVID-19) pandemic have resulted in increasingly complex patient populations and working environments (Academy of Science of South Africa [ASSAF] 2018; Fawaz et al. 2018). Of concern is the health and well-being of university students with elevated levels of stress, depression, burnout, and suicidality reported, especially by healthcare students (Brewer et al. 2019; Isaacs 2019; Smith et al. 2021).

Work integrated learning seems particularly challenging for students as they tend to struggle to manage day-to-day encounters in the clinical environment (Probst et al. 2014; Smith et al. 2021). In addition, tertiary institutions are faced with student populations that are diverse in gender, race, social standing, geographical origin and religious orientation (ASSAf 2018; Chen 2017; Fawaz et al. 2019). Therefore, students encounter different cultural groups and groups of peers who behave and interact differently than those familiar to them. Through cultural and interpersonal adaptability, cultural competence is thus required for positive health outcomes (Matthews & Van Wyk 2018) and to function optimally in diverse work and educational environments. According to Rowe (2015), to function successfully within a complex and ever-changing clinical environment healthcare students and professionals must be able to adapt to a dynamic environment and continuously improve their performance and skills rather than just competency.

Adaptive performance is defined as ‘an individual’s ability to adapt to dynamic work-related situations by modifying their behaviour according to the requirements of the new environment, situations, or events’ (Charbonnier-Voirin & Roussel 2012:281). Changes in the work environment may arise from organisational reorganisation, changed priorities or limited resource availability, requiring individuals to adapt quickly and make decisions during a time of uncertainty. Adapting in the face of a constantly changing healthcare environment is an essential skill of successful healthcare professionals (Fawaz et al. 2018) and if training institutions wish to continue graduating employable graduates, fostering adaptive performance is essential. Pulakos et al. (2000:620) described eight dimensions of adaptive performance including dealing with unpredictable and uncertain work situations, handling of emergencies and crisis situations, creative problem solving, handling work stress, learning of new tasks, technologies and procedures, as well as demonstrating interpersonal, cultural and physically orientated adaptability. Successful adaptive performance implies that an individual can manage uncertain and unpredictable work situations efficiently (Charbonnier-Voirin & Roussel 2012).

With the arrival of the Fourth Industrial Revolution (4IR), healthcare educators have been faced with the task of adjusting their educational strategies to ensure graduate employability (Van der Merwe 2021) through equipping them with essential attributes, such as teamwork, problem-solving skills, critical thinking, improved communication and leadership capabilities, to enable them to manage the multifaceted dynamics of modern-day healthcare (Daggett 2017; Hussin 2018; World Bank 2018). In addition, to ensure graduate employability the integration and attainment of graduate attributes related to teamwork, a person-centred care approach and effective communication is vital (Vogenberg & Santilli 2018; Griesel & Parker 2009; World Bank 2018). Graduate employability capacities, healthy self-esteem, and adaptability in challenging situations as well as in new challenges have been identified as essential attributes in developing skilled and employable graduates (Chetty 2012; Coetzee, Ferreira & Potgieter 2015).

The CanMEDS framework is one of the frameworks describing the multiple roles, including various competencies within each role, of a healthcare professional (Frank & Danhoff 2007). A traditional approach to teaching and learning of healthcare professional students is structured to specifically emphasise the development of the above-mentioned competencies (Fraser & Greenhalgh 2001), but according to Jarvis-Selinger, Pratt and Regehr (2012), using these competencies can potentially oversimplify the role of the healthcare professional. If healthcare graduates can develop the ability of adapting to an ever-changing and complex healthcare system, they also need to develop the self-assessment skills to identify their own personal learning needs before they can improve their own practice (Colthart et al. 2008). The challenge for educators and clinicians is in appreciating the intricacy of developing the adaptability of undergraduate students and emphasis should be placed on developing this vital skill (Rowe 2015).

A familiar concept in healthcare research is resilience. Resilience is integral to students’ mental health and success both in the institutional setting and the workplace (Brewer et al. 2019; Sanderson & Brewer 2017). Resilience is described by Brewer et al. (2019:1115) as ‘either a process or an outcome in relation to positive adaptation.’ It is not surprising that one of the key traits of a resilient healthcare professional identified by Matheson et al. (2016) is the ability to be adaptable. Considering the definition of adaptive performance provided earlier in this article (Charbonnier-Voirin & Roussel 2012), we view resilience and adaptive performance as interwoven concepts. Most graduate attributes expected by the Health Professions Council of South Africa for physiotherapy students have been identified as the key traits required for a resilient healthcare professional (Matheson et al. 2016). We suggest that resilience may be viewed as an umbrella term under which the graduate attributes fall. To foster resilience, an individual is required to develop the ability to adapt to changing situations. According to Ismail (2017), young South Africans must be more adaptable to change in the working environment, a comment which still seems relevant today. Considering the competencies expected of healthcare graduates (Daggett 2017; Hussin 2018; World bank 2018), a concern raised by the National Physiotherapy Educator’s Forum (2019) is that South African physiotherapy graduates seem to demonstrate a lack of the aforementioned skills. Therefore, adaptive performance is vital for healthcare professionals as it involves managing changing environments and stressors, generating unique or innovative ideas, employing creative solutions to solve problems, and performing when resources are inadequate. No research is available in South Africa regarding the adaptive performance of physiotherapy students. Our study aimed to investigate the adaptive performance of final year undergraduate physiotherapy students at the University of the Free State.

## Methods

A quantitative descriptive study was performed.

No sampling method was utilised as all consenting final year undergraduate physiotherapy students registered at the UFS in 2021 (*n* = 28) were approached by us for inclusion. An information leaflet detailing the purpose and procedure of our study was electronically provided to the targeted population at the end of their 2021 academic year (October 2021). Participants were made aware that informed consent was implied upon completing the standardised questionnaire. The short 55-item I-ADAPT measurement (I-ADAPT-M) was imported into Google Forms: a survey administration software offered by Google. The questionnaire link was distributed to participants utilising their official registered university email address. Participants had 2 weeks to complete the questionnaire, which took approximately 10 minutes to complete, with a follow-up reminder sent weekly during the data collection period. As a result of the online nature of our study, COVID-19 safety protocols did not influence our study procedures, and participants remained unknown to each other but not to us, allowing for unhindered responses, and follow-up reminders for questionnaire completion could be distributed easily.

The I-ADAPT-M, developed and validated by Ployhart and Bliese (2006), is based on the eight dimensions of adaptive performance proposed by Pulakos et al. (2000). The 55 items included in the I-ADAPT-M were developed using definitions of each of the above-mentioned eight dimensions (Murphy 2015; Ployhart & Bliese 2006). Each item is rated on a 5-point Likert scale, including options for strongly disagree, disagree, neutral, agree, and strongly agree. A summary of the number of statements per dimension and the scoring of each dimension is indicated in Table 1.

**TABLE 1:** Scoring of the I-ADAPT measurement.

Dimension	Number of statements	Scoring
Demonstrating cultural adaptability	5	No reverse scored statements
Demonstrating physically orientated adaptability	9	Four reverse scored statements
Demonstrating interpersonal adaptability	7	No reverse scored statements
Learning new tasks, technologies and procedures	9	No reverse scored statements
Handling work stress	5	All statements reverse scored
Creative problem solving	5	No reverse scored statements
Handling emergencies and crisis situations	6	No reverse scored statements
Handling unpredictable and uncertain work situations	9	Two reverse scored statements

*Source:* Adapted from Ployhart, R.E. & Bliese, P.D., 2006, ‘Individual Adaptability (IADAPT) Theory: Conceptualizing the antecedents, consequences, and measurement of individual differences in adaptability’, in C. Shawn Burke, L.G. Pierce & E. Salas (eds.), *Understanding adaptability: A prerequisite for effective p erformance within complex environments (Advances in human performance and cognitive engineering research, Vol. 6)*, pp. 3–39, Emerald Group Publishing Limited, Bingley

Each of the eight dimensions was analysed as individual data sets. No normative values are available, as adaptability is viewed as an individual process in response to change and therefore develops and unfolds over time (Baard, Rench & Kozlowski 2014). A high score on the eight adaptability dimensions included in the I-ADAPT-M is viewed as the participant having increased adaptive performance (Baard et al. 2014).

As a result of the nature of the statements posed to students, emotional reactions may have been elicited from participating students; therefore, the contact details of student counselling at the University of the Free State were made available to participants to schedule an appointment with a trained psychologist free of charge if the need arose.

### Data analysis

Descriptive statistics were utilised. Frequencies and percentages were calculated for categorical data, with medians and interquartile ranges calculated for numerical data. Each dimension was calculated individually, and reversed scoring was applied as indicated by the questionnaire (Table 1).

The ‘handling emergencies and crisis situations’ dimension consisted of six questions with a maximum score of 30. Both the ‘demonstrating cultural adaptability’ and ‘handling work stress’ dimensions had five questions with a maximum score of 25. Seven questions were posed in the ‘demonstrating interpersonal adaptability’ dimension, and the maximum score was 35. Nine questions, with a maximum score of 45 for each dimension, were included in the ‘learning new tasks, technologies and procedures’, ‘demonstrating physically orientated adaptability’, and ‘handling unpredictable and uncertain work situations’ dimensions. A total of five questions were posed to participants for ‘creative problem solving’ with a maximum score of 25.

Although Google Forms reports data in Microsoft Excel, avoiding input errors, the first author manually checked the data to ensure that no errors occurred during the data clean-up process. The first two authors independently reviewed the data to ensure unbiased reporting.

### Ethical considerations

Our study commenced following ethical approval by the Health Sciences Research Ethics Committee and relevant authorities at the University of the Free State (UFS-HSD2021/0349/2906).

## Results

The response rate for our study was 28.5% (*n* = 8), with all forms completed in full. The data were not normally distributed, and therefore the values are reported as median and interquartile ranges. Median scores per dimension are presented in Table 2, with the work stress dimension scoring the lowest with a median score of 12.5 out of a maximum of 25.

**TABLE 2:** Numerical scores per dimension.

Dimension (maximum score)	*n*	Median	Lower quartile	Upper quartile	Minimum	Maximum
Uncertainty (45)	8	28.0	27.0	35.5	26.0	41.0
Creativity (25)	8	16.0	14.5	19.0	14.0	22.0
Physical (45)	8	34.5	32.5	35.0	31.0	41.0
Learning (45)	8	36.0	35.0	39.5	30.0	43.0
Interpersonal (35)	8	30.0	29.5	32.0	27.0	35.0
Work stress (25)	8	12.5	10.5	16.5	8.0	24.0
Cultural (25)	8	21.0	18.5	23.5	18.0	25.0
Crisis (30)	8	24.0	22.0	27.0	20.0	29.0

*Source:* Adapted from Ployhart, R.E. & Bliese, P.D., 2006, ‘Individual Adaptability (IADAPT) Theory: Conceptualizing the antecedents, consequences, and measurement of individual differences in adaptability’, in C. Shawn Burke, L.G. Pierce & E. Salas (eds.), *Understanding adaptability: A prerequisite for effective p erformance within complex environments (Advances in human performance and cognitive engineering research, Vol. 6)*, pp. 3–39, Emerald Group Publishing Limited, Bingley

Uncertainty, unpredictable and uncertain work situations; Creativity, creative problem solving; Physical, demonstrating physically orientated adaptability; Learning, learning of new tasks, technologies and procedures; Interpersonal, demonstrating interpersonal adaptability; Work stress, handling work stress; Cultural, demonstrating cultural adaptability; Crisis, handling of emergencies and crisis situations.

As highlighted in bold in Table 3, the dimensions that scored the lowest are the dimensions related to uncertainty (62.2%), creativity (64.0%) and work stress, which scored the lowest with 50.0%. The highest scores were achieved in the dimensions pertaining to the demonstration of interpersonal (85.7%) and cultural adaptability (84%).

**TABLE 3:** Percentage scores per dimension.

Dimension	*n*	Median %	Lower quartile %	Upper quartile %	Minimum %	Maximum %
Uncertainty	**8**	**62.2**	**60.0**	**78.9**	**57.8**	**91.1**
Creativity	**8**	**64.0**	**58.0**	**76.0**	**56.0**	**88.0**
Physical	8	76.7	72.2	77.8	68.9	91.1
Learning	8	80.0	77.8	87.8	66.7	95.6
Interpersonal	8	85.7	84.3	91.4	77.1	100.0
Work stress	**8**	**50.0**	**42.0**	**66.0**	**32.0**	**96.0**
Cultural	8	84.0	74.0	94.0	72.0	100.0
Crisis	8	80.0	73.3	90.0	66.7	96.7

*Source:* Adapted from Ployhart, R.E. & Bliese, P.D., 2006, ‘Individual Adaptability (IADAPT) Theory: Conceptualizing the antecedents, consequences, and measurement of individual differences in adaptability’, in C. Shawn Burke, L.G. Pierce & E. Salas (eds.), *Understanding adaptability: A prerequisite for effective performance within complex environments (Advances in human performance and cognitive engineering research, Vol. 6)*, pp. 3–39, Emerald Group Publishing Limited, Bingley

Note: Data in bold reflects lowest scoring dimensions.

Inconsistent statements relating to participants’ inability to adapt to unpredictable and uncertain work situations were found. Most participants (75%) agreed that they are ‘able to make effective decisions without all relevant information’; however, 62.5% of participants indicated that they ‘become frustrated when things are unpredictable’. Although 75% of participants indicated that they tend to perform best in stable conditions, all the participants (100%) agreed that they are ‘able to adapt to changing situations’ and can ‘adjust my plans to changing conditions’. Only 50% of the participants agreed that they ‘perform well in uncertain situations’, with 37.5% of participants agreeing that they ‘need for things to be black and white’. Interestingly, 62.5% of participants indicated that they can ‘easily respond to changing conditions’. Most of the participants (87.5%) agreed that they ‘can readily change gears’ when something unexpected happens.

Most participants (62.5%) experienced challenges with managing work stress, most notably in managing a large workload (75%) (Table 4).

**TABLE 4:** Handling work stress (*n* = 8).

Question number	Question posed	Response		
		Agree (%)	Neutral (%)	Disagree (%)
3	I usually over-react to stressful news.	37.5	12.5	50.0
15	I feel unequipped to deal with too much stress.	62.5	0.0	37.5
21	I am easily rattled when my schedule is too full.	37.5	12.5	50.0
32	I am usually stressed when I have a large workload.	75.0	0.0	25.0
35	I often cry or get angry when I am under a great deal of stress.	62.5	12.5	25.0

*Source*: Adapted from Ployhart, R.E. & Bliese, P.D., 2006, ‘Individual Adaptability (IADAPT) Theory: Conceptualizing the antecedents, consequences, and measurement of individual differences in adaptability’, in C. Shawn Burke, L.G. Pierce & E. Salas (eds.), *Under standing adaptability: A prerequisite for effective performance within complex environments (Advances in human performance and cognitive engineering research, Vol. 6),* pp. 3–39, Emerald Group Publishing Limited, Bingley

Interestingly, a quarter to a third of participants selected neutral to the questions posed, possibly indicating that they are unsure regarding their creative problem-solving abilities (Table 5).

**TABLE 5:** Creative problem solving (*n* = 8).

Question number	Question posed	Response		
		Agree (%)	Neutral (%)	Disagree (%)
10	I see connections between seemingly unrelated information.	62.5	25.0	12.5
16	I am good at developing unique analyses for complex problems.	62.5	25.0	12.5
24	I am an innovative person.	37.5	25.0	37.5
36	When resources are insufficient, I thrive on developing innovative solutions.	37.5	37.5	25.0
37	I am able to look at problems from a multitude of angles.	50.0	37.5	12.5

*Source*: Adapted from Ployhart, R.E. & Bliese, P.D., 2006, ‘Individual Adaptability (IADAPT) Theory: Conceptualizing the antecedents, consequences, and measurement of individual differences in adaptability’, in C. Shawn Burke, L.G. Pierce & E. Salas (eds.), *Under standing adaptability: A prerequisite for effective performance within complex environments (Advances in human performance and cognitive engineering research, Vol. 6)*, pp. 3–39, Emerald Group Publishing Limited, Bingley

Participants performed best in the dimension relating to interpersonal adaptability, with 100% agreeing with five questions and 87.5% agreeing with the remaining two questions, respectively (Table 6).

**TABLE 6:** Interpersonal adaptability (*n* = 8).

Question number	Question posed	Response		
		Agree (%)	Neutral (%)	Disagree (%)
4	I believe it is important to be flexible in dealing with others.	100.0	0.0	0.0
7	I tend to be able to read others and understand how they are feeling at any particular moment.	87.5	12.5	0.0
18	My insight helps me to work effectively with others.	100.0	0.0	0.0
30	I am an open-minded person in dealing with others.	100.0	0.0	0.0
33	I am perceptive of others and use that knowledge in interactions.	100.0	0.0	0.0
42	I try to be flexible when dealing with others.	100.0	0.0	0.0
50	I adapt my behaviour to get along with others.	87.5	12.5	0.0

*Source*: Adapted from Ployhart, R.E. & Bliese, P.D., 2006, ‘Individual Adaptability (IADAPT) Theory: Conceptualizing the antecedents, consequences, and measurement of individual differences in adaptability’, in C. Shawn Burke, L.G. Pierce & E. Salas (eds.), *Under standing adaptability: A prerequisite for effective performance within complex environments (Advances in human performance and cognitive engineering research, Vol. 6)*, pp. 3–39, Emerald Group Publishing Limited, Bingley

Most (75%) participants indicated that they cannot work in a disorganised environment, with 50% indicating that they require a comfortable environment to perform well. Overall, participants agreed with statements pertaining to their acceptance of learning new tasks, technologies, and procedures and their perceived cultural adaptability. However, 62.5% of participants indicated that they have a neutral outlook on their enjoyment of learning regarding cultures other than their own.

## Discussion

Our study results indicated that undergraduate physiotherapy students at the University of the Free State perceive themselves as being able to demonstrate adaptive performance. The lack of normative values makes result interpretation challenging, as adaptive performance is viewed as an individual process that develops over time (Baard et al. 2014).

Of concern is the poor scoring of participants in the dimension relating to the handling of work stress. Challenges in the workplace, including undergraduate studies, may have various sources, including information overload, time pressures, and workload (Matheson et al. 2016). The interpersonal nature of healthcare is also a significant source of stress for healthcare professionals, notably because of emotional demands, long working hours, and complex and challenging work environments (Foster et al. 2018; Matheson et al. 2016; Probst et al. 2014; Smith et al. 2021). Many participants reported that they require a comfortable and orderly work environment to function optimally, which is not always possible because of diverse healthcare settings, varied resources, and the nature of healthcare practice (Matheson et al. 2016). Smith et al. (2021) recommend that students are equipped with skills on how to manage stress and large workloads and how to navigate uncomfortable and unfamiliar situations. At an undergraduate level, students should be exposed to a variety of environments to sensitise them to unfamiliar situations and debriefing should take place regularly to assist students in becoming comfortable with the uncomfortable (Cheng et al. 2018; Smith et al. 2021). Students should be encouraged to reflect, be mindful, and recognise their own limitations (Smith et al. 2021) and acknowledge that their own personal preferences and personal views may impede their professional role (Delany et al. 2015). Uncomfortable situations or stressful encounters should be embedded in already existing learning and teaching activities thereby not overburdening staff and students (Smith et al. 2021). As a result of the seemingly high prevalence of work stress experienced by undergraduate physiotherapy students, trained facilitators could present scheduled interventions (Smith et al. 2021) for students to provide them with strategies on how to manage stressful and uncomfortable situations not only in the clinical areas but also in class situations. We acknowledge academic pressures on both staff and students and therefore advise an interprofessional approach to such interventions facilitated by experts in the field.

Within healthcare practice, uncertainty and unpredictability are unavoidable, with studies identifying a lack of strategies on the part of students to deal with day-to-day challenges in a clinical environment (Probst et al. 2014). One aspect of adaptability involves acting without detailed information and adjusting one’s cognition and behaviour when necessary. It has been described in the literature that individuals who demonstrate improved adaptability are more willing to participate in uncertain situations and have previously exposed themselves to a broader range of experiences, thereby enabling themselves to ‘practice’ different approaches to execute and adapt to tasks. Increased exposure to various experiences provides a greater repertoire from which to draw and increase adaptability (Lepine, Colquitt & Erez 2000). Although participants agreed that they performed best in stable situations, they reported that they can adjust when the situation required it. A reason for this might be that at the time of our study, these students were already used to managing unpredictable academic and personal situations resulting from the COVID-19 pandemic. Considering that employers prefer healthcare workers who can function independently and as part of a team in a wide range of clinical environments (Fawaz et al. 2018), undergraduate teaching should build on their curricula to maintain the output of students who can adjust in unpredictable and uncertain situations.

As described in a study by Murphy (2015), the components of adaptation include a changing task, recognition of the cues indicating the need to change, altering one’s cognitions, affect, or behaviour, and success after the change. A contribution of the aforementioned conceptualisation is that the change does not have to be purely behavioural but can also be cognitive or emotional. In line with this argument, emotional intelligence refers to a person’s ability to effectively distinguish, understand, and manage the emotions of both self and others and is essential for healthcare professionals in navigating their practice (Foster et al. 2018). With participants indicating that they tend to react emotionally when under stress or faced with uncertain and unpredictable situations, embedded educational interventions strengthening emotional intelligence are essential. It has been shown that increased emotional intelligence positively affects perceived student stress (Foster et al. 2018).

Problem-solving ability is an essential graduate attribute, especially creative problem-solving because of challenging healthcare settings, patients, and resources. Self-reported results from our study indicated that participants deemed themselves equipped to solve problems creatively. However, several participants indicated they merely felt neutral regarding their creative problem-solving skills. Although a neutral answer does not imply that a student does not possess the ability, it indicates uncertainty. Fostering creative problem-solving skills practically is essential and real-world scenarios should be enacted for students to develop confidence in this skill.

Participants responded positively concerning their acceptance of learning new tasks and procedures, using new technologies, as well as their perceived cultural adaptability. This may be because of the increasingly diverse educational activities and the peers students encounter (ASSAf 2018; Chen 2017; Fawaz et al. 2019; Matthews & Van Wyk 2018). However, more than half of participants reported a neutral outlook on their enjoyment of learning regarding cultures other than their own. Matthews and Van Wyk (2018) reported on the need expressed by medical students at a South African university to include additional cultural competence training in their curriculum. This need might be seated in a feeling of uncertainty and unpredictability regarding their view of their cultural competence, and we hypothesise that this might have also contributed to participants in our study remaining in a neutral space with regard to their acceptance of learning of other cultures.

It is no longer sufficient that young adults have a degree in modern society, but it is also required that they must exhibit and apply a combination of desired qualities and practices effectively in the working environment. (Hogan, Chamorro-Premuzic & Kaiser 2013; Stevenson & Clegg 2011; Yorke & Knight 2006). The I-ADAPT theory states that adaptive performance depends on an individual’s knowledge, skills, and abilities and, as a result, predicts task and contextual performance. Knowledge acquisition, coping strategy selection, and situation appraisal mediate the process of developing adaptive performance. Creating an environment where students feel like valued members of the institution and can develop personal traits such as goal orientation, self-management, and resourcefulness is not only important for student retention (Barbatis 2010) but also in fostering the development of resilience (Matheson et al. 2016; Smith et al. 2021) and thereby adaptability. The future of many healthcare professions lies in the adaptability of graduates and creative and innovative educators to foster essential skills in a new generation.

### Limitations

Our study results may not be generalisable due to the small sample size of the study. However, the results obtained warrant further investigation into the adaptive performance of undergraduate physiotherapy students. As the I-ADAPT-M consists of closed-ended questions, clarification by means of participant comments was not possible. Considering the increasing demand for student feedback (Porter, Whitcomb & Weitzer 2014) over-surveying of students may have contributed to the low response rate of our study. Other forms of data collection could have been considered, an example being telephonic completion of questionnaires.

## Conclusion

Uncertainty and unpredictability are inevitable in a healthcare setting – both during undergraduate and clinical practice. Participants indicated that they experienced challenges with handling work stress and often experienced emotional reactions when they experienced stress and unpredictable situations. Although participants agreed that they performed better in stable and comfortable environments, they indicated that they feel they can adjust to changing situations.

Future studies are proposed focussing on identifying the potential need for educational interventions focussing on stress management and emotional intelligence development and should be included in undergraduate programmes. Student support structures should foster student confidence that they will succeed if they embrace the challenges and unfamiliar and uncomfortable situations.
